# Patient Experience and Feasibility of a Remote Monitoring System in Parkinson's Disease

**DOI:** 10.1002/mdc3.14169

**Published:** 2024-07-26

**Authors:** Bart R. Maas, Daniël H.B. Speelberg, Gert‐Jan de Vries, Giulio Valenti, Andreas Ejupi, Bastiaan R. Bloem, Sirwan K.L. Darweesh, Nienke M. de Vries

**Affiliations:** ^1^ Department of Neurology, Radboud University Medical Center, Donders Institute for Brain, Cognition and Behavior Center of Expertise for Parkinson and Movement Disorders Nijmegen The Netherlands; ^2^ Philips Research—Healthcare Eindhoven The Netherlands

**Keywords:** Parkinson's disease, wearable sensors, remote monitoring, feasibility, experience

## Abstract

**Background:**

Remote monitoring systems have the potential to measure symptoms and treatment effects in people with Parkinson's disease (PwP) in the home environment. However, information about user experience and long‐term compliance of such systems in a large group of PwP with relatively severe PD symptoms is lacking.

**Objective:**

The aim was to gain insight into user experience and long‐term compliance of a smartwatch (to be worn 24/7) and an online dashboard to report falls and receive feedback of data.

**Methods:**

We analyzed the data of the “Bringing Parkinson Care Back Home” study, a 1‐year observational cohort study in 200 PwP with a fall history. User experience, compliance, and reasons for noncompliance were described. Multiple Cox regression models were used to identify determinants of 1‐year compliance.

**Results:**

We included 200 PwP (mean age: 69 years, 37% women), of whom 116 (58%) completed the 1‐year study. The main reasons for dropping out of the study were technical problems (61 of 118 reasons). Median wear time of the smartwatch was 17.5 h/day. The online dashboard was used by 77% of participants to report falls. Smartphone possession, shorter disease duration, more severe motor symptoms, and less‐severe freezing and balance problems, but not age and gender, were associated with a higher likelihood of 1‐year compliance.

**Conclusions:**

The 1‐year compliance with this specific smartwatch was moderate, and the user experience was generally good, except battery life and data transfer. Future studies can build on these findings by incorporating a smartwatch that is less prone to technical issues.

Parkinson's disease (PD) is the second‐most common neurodegenerative disease globally, and the number of affected individuals has more than doubled over the past 3 decades.^1^, ^2^ Adequate treatments, such as well‐timed medication, treatment by allied health professionals, and lifestyle measures such as exercise, can alleviate burdensome symptoms for many people with PD (PwP). However, clinical decisions are largely based on relatively brief in‐clinic evaluations that are often not representative of functioning in daily life. Objective data of a person's functioning during the day in his or her own home environment, with insight into, for example, physical activity, falls, sleep problems, or the response to medication, may provide valuable additional insights to both clinicians and PwP, thereby promoting personalized healthcare.^3^, ^4^, ^5^ Remote monitoring also offers important opportunities by yielding digital progression biomarkers that can serve as exploratory outcomes in clinical trials that aim to test the efficacy of new symptomatic or disease‐modifying treatments for PwP. Before implementation of such remote monitoring systems in clinical trials or in daily practice, their feasibility, including compliance, should be investigated in a representative population of PwP.

Only a few studies investigated the compliance of remote monitoring systems in PwP.^6^, ^7^, ^8^, ^9^, ^10^ These studies had a small sample size,^7^, ^9^, ^10^, ^11^, ^12^ a short follow‐up duration,^8^, ^9^, ^10^, ^11^, ^12^ or PwP with relatively mild symptoms.^8^ A common finding of these feasibility studies was a substantial dropout of study participants, with dropout rates increasing over time.^7^, ^8^, ^10^ Reasons for dropout included technical issues, complexity of the deployed systems, decrease in interest after the novelty had worn off, study fatigue, and a lack of feedback to the participants. However, these disadvantages may well depend on the specific device at hand in each of these studies.

Furthermore, it is unknown which demographic and clinical characteristics contribute to higher satisfaction and better compliance. Such information is warranted to gain insight into whether there are subgroups of PwP in whom such remote monitoring systems are more feasible than in other subgroups. Therefore, the aim of this study was to describe the experiences of PwP with a remote monitoring system that consisted of a smartwatch as well as an online dashboard that provided insight into physical activity and fall pattern measured using the smartwatch. Additionally, we endeavor to gain more insight into participant characteristics to better understand determinants of compliance.

## Patients and Methods

### Study Design and Setup

**FIG. 1:**
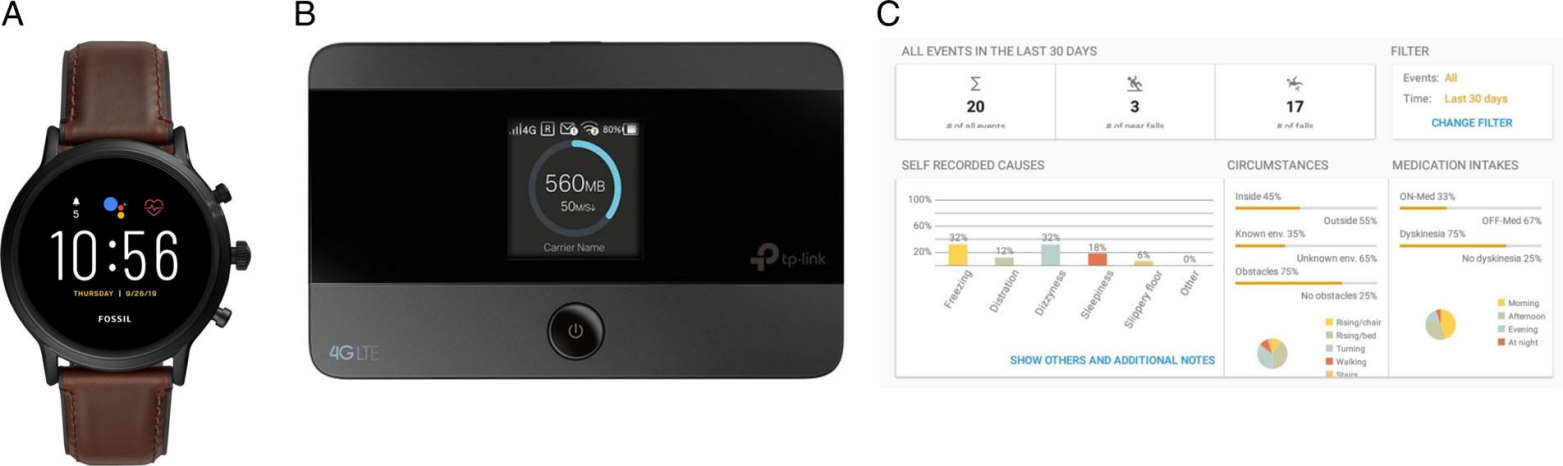
Study setup. The setup of our remote monitoring system. Participants wore (**A**) a wearable sensor (Fossil Carlyle Gen 5 FTW4026) that automatically collected continuous data regarding physical activity. Data were automatically transferred to our server during charging of the wearable sensor using (**B**) the WiFi Hub. In addition, participants reported their falls either on (**C**) an online dashboard by selecting a registered fall or adding a new fall or in a paper‐based diary. Participants can view their physical activity and falls using this online dashboard.

We conducted an observational cohort study entitled “Bringing Parkinson Care Back Home” (ClinicalTrials.gov ID NCT04288583) where PwP were followed up for 1 year using a remote monitoring system. The aim of this study was to use a remote monitoring system to gain insight into the patterns of falls and physical activity over 1 year, to study the association between them, and to identify predictors for both variables in a population with an increased fall risk. The primary outcome of this will be reported separately; we here focus on compliance issues. The setup of our specific remote monitoring system is shown in Figure 1. Participants wore a smartwatch (Fossil Carlyle Gen 5 FTW4026) (Fig. 1A) that contained an accelerometer and a gyroscope. The specific instruction to the participants was to wear the smartwatch as long as possible throughout the day (also during the night and showers), except for moments when the watch had to be charged and data had to be transferred.

### Participants

Participants were recruited through the outpatient clinic of the Radboud University Medical Centre, the ParkinsonNet network^13^ (Dutch nationwide network of healthcare professionals specialized in Parkinson care), ParkinsonNEXT^14^ (online Dutch platform that connects PwP and researchers), and the PRIME‐NL study^15^ (ongoing study at the Radboud University Medical Center, Nijmegen, the Netherlands). PwP within these sources are representative of the full spectrum of PwP in the Netherlands in terms of demographic characteristics. Inclusion ran from November 2021 until June 2022, and participants were eligible if they confirmed a diagnosis of PD, were 18 years old or older, were fluent in Dutch, and were willing and able to provide informed consent. Furthermore, participants should have experienced at least 1 fall incident in the previous 6 months. Participants were excluded if they exhibited cognitive or psychiatric impairments that might hinder completion of the study. When subjects chose to leave the study, we recorded the reason for dropout.

### Procedure and Assessments

The study procedure started with telephone screening of participants based on inclusion criteria, after which an appointment was made for a home visit. During the home visit, informed consent was signed, and several clinical tests were performed for the primary aim of the Bringing Parkinson Care Back Home study. The Wi‐Fi hub was set up by a team member to automatically transfer data (Fig. 1B), and participants were instructed how to charge the watch independently themselves. In addition, participants were trained on how to report their falls either using an online dashboard (WebApp) (Fig. 1C) by selecting a registered fall or by adding a new fall or using a paper‐based diary. Participants received a weekly reminder by email to complete this fall diary. In this online dashboard, participants were able to gain insight into their own physical activity and fall patterns if they were interested in this. During office hours, a helpdesk was available by telephone or email to support with technical problems. Furthermore, researchers contacted the participants at least twice during the study year to ask whether the participants had any questions. Additionally, the participants were proactively called to offer technical support when we received no sensor data for 4 consecutive weeks. This was done because sensor data could be stored for approximately 6 weeks on the smartwatch without transferring to the server, before losing sensor data.

At baseline, motor symptom severity (Unified Parkinson's Disease Rating Scale, Part III [UPDRS‐III]), walking speed (6‐meter walking test), balance (Mini‐Balance Evaluation Systems Test [miniBEST]), and cognition (Montreal Cognitive Assessment [MoCA]) were assessed by a well‐trained research assistant of the Radboud University Medical Centre during a home visit. Demographic characteristics, mentation, behavior and mood (UPDRS, Part I), activities of daily living (UPDRS, Part II), complications of therapy (UPDRS, Part IV), physical activity (LASA physical activity questionnaire [LAPAq]), freezing (new freezing of gait q), fatigue (fatique severity scale [FSS]), anxiety and depression (hospital anxiety and depression scale [HADS]), and quality of life (parkinson's disease questionnaire‐ 39 [PDQ‐39]) were assessed using questionnaires at baseline. After completion of follow‐up at 12 months, questionnaires were repeated. These questionnaires at follow‐up also included a questionnaire with structured answer options regarding experience of study and smartwatch, which were only filled in by participants who completed the 1‐year study period (Supplementary Material B).

### Analyses

First, baseline characteristics, duration of study participation, and reason for dropout were analyzed using descriptive statistics.

Second, we evaluated the user experience regarding the smartwatch of participants who successfully completed the 1‐year study period.

Finally, we employed multiple Cox regression models to identify both demographic and clinical characteristics that were associated with study duration of participants. Our analyses of associations between characteristics and study duration were exploratory, and therefore we assume coefficients with a *P*‐value of <0.05 as a significant association. All participants were included in the described analyses, but to account for people who did not choose to drop out from the study, we added a sensitivity analysis in which we removed participants who received a diagnosis of atypical parkinsonism or who died during the study period.

Regression analyses were performed in *R* using the *survival* package.

## Results

### Sociodemographic Characteristics

Sociodemographic characteristics are presented in Table 1. We included 200 PwP with a mean age of 69 years (standard deviation = 7.4). A total of 72 participants were women (37%). On average, participants had a disease duration of 9.1 years, and most participants had a disease severity (Hoehn & Yahr [H&Y]) of stage 2 or 3 (79%). Of the 200 participants, 15 did not return their baseline questionnaires, which resulted in missing data on clinical/demographic characteristics in these participants.

**TABLE 1:** Sociodemographic characteristics

Characteristic	Study cohort n = 200
Age (yr)^a^	68.7 (7.4)
Women, n (%)	74 (37)
Education, n (%)^b^	
None	2 (1)
Secondary school	28 (15)
Lower vocational education	17 (9)
Secondary vocational education	38 (21)
Higher professional education	67 (37)
University	31 (17)
Disease characteristics	
Years since diagnosis^b^	9.1 (5.8)
Hoehn & Yahr scale, n (%)^c^	
I	12 (6)
II	65 (33)
III	94 (47)
IV	27 (14)
V	1 (<1)

Values are mean (standard deviation), unless stated otherwise. Missing values are excluded in calculation percentages.

^a^ Missing data of 15 participants.

^b^ Missing data of 17 participants.

^c^ Missing data of 1 participant.

### Compliance

**FIG. 2:**
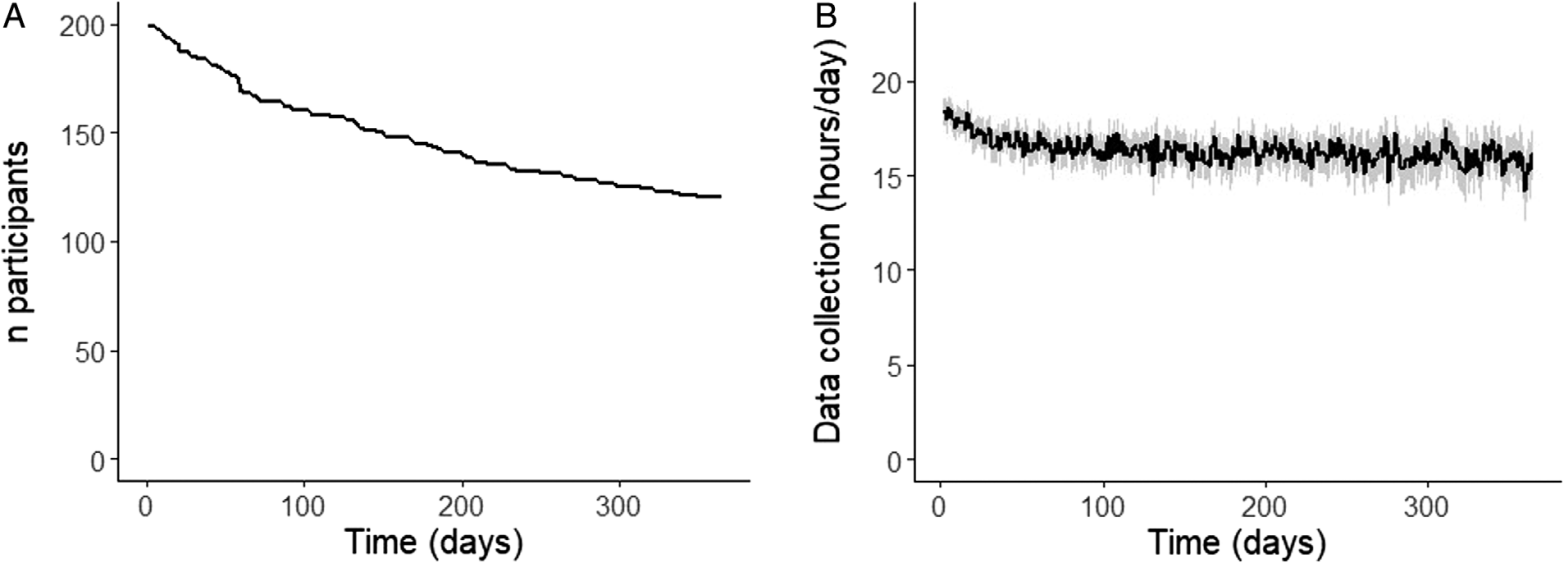
Compliance. (**A**) The number of participants over the study period of 365 days. (**B**) The mean hours of data collection per day in these participants are indicated by the black line. The gray area in (B) shows the 95% confidence intervals.

Of the 200 PwP who were included, 84 participants (42%) dropped out during the study period (Fig. 2A). Average duration of study participation was 266 days, which was capped by the maximum study duration of 1 year. In 4 participants, the clinical diagnosis of PD was changed to a diagnosis of atypical parkinsonism during the study period (these participants remained in the study), and 2 participants died.

The median wear time of the smartwatch was 17.5 h/day (Fig. 2B). Over the year, this duration declined from a median wear time of 18.8 h in the 1st month to 16.1 h in the 12th month.

### Reasons for Dropout

**FIG. 3:**
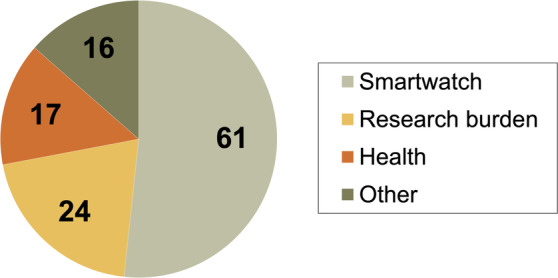
Reasons for dropout. Frequency of reasons for dropout is categorized (84 participants, 118 reasons). Participants were able to give multiple reasons (also that fall into the same category). Exact reasons for dropout are presented in Supplementary Material A.

The most frequently cited reason for dropping out was problems experienced with the watch (n = 61), including a short battery life (n = 18), or technical problems (n = 22), including problems with data transfer (Fig. 3; Supplementary Material A). Other reasons for dropping out of the study were related to research burden (n = 24) or health issues such as increase in severity of PD (n = 17). Reasons for dropout did not change substantially between the first 3 months and the last 3 months of the study (48% vs. 42% smartwatch related, respectively; 21% vs. 17% research burden related, respectively; 14% vs. 17% health related, respectively; and 16% vs. 25% miscellaneous, respectively).

### Technical Support

Of all 200 participants, 102 received at least 1 new smartwatch during the study period because of technical problems with their original smartwatch. In total 133 watches were replaced (range: 1–4 per participant). No response of the smartwatch (n = 48) and short battery life (n = 43) were most common reasons to replace the smartwatch. Other reasons were no data transfer or application for data transfer removed (n = 13), allergic reaction to the strap (n = 10), and other less‐frequently cited reasons such as unintentional change of time and date (n = 4) or a broken strap (n = 1).

Of the 84 participants who dropped out of the study, 32 participants (38%) received at least 1 new watch, and in total 43 watches were replaced in this subgroup. Of the 116 participants who completed the study, 70 participants (60%) received at least a new watch, and in total 90 watches were replaced in this subgroup.

### Study Experience and Technical Support

Of the 116 participants who completed the study year, 98 participants (84%) filled in a questionnaire regarding their experience of the sensor and the study (Supplementary Material B). Demographic and clinical characteristics of those 98 participants are comparable to the full study population (Supplementary Material D). All the 98 participants considered both the home visit and the telephone contact moments with the research team as neutral to very pleasant. For the majority of participants, watch appearance, size, size of icons, and strap comfort were acceptable, and 77% of participants who filled in the questionnaire would participate in another study with a similar device.

A total of 25 participants experienced issues that limited the wearing of the smartwatch, of which a short battery life was the most frequently cited reason (in 18 of 25 participants).

The online dashboard was sometimes or regularly used by 77% of participants filling in the questionnaire. Only 16 participants visited the online dashboard to gain more insight into their own movement pattern, of whom only 3 indicated to have adjusted their lifestyle.

### Association between Demographic and Clinical Characteristics and Duration of Study Participation

Results regarding the association between demographic and clinical characteristics and duration of study participation are presented in Table 2. In our sample, we found that people who did possess a smartphone were twice as likely to complete the 1‐year study (Hazard Ratio [HR] = 0.49, *P* = 0.01). Furthermore, a lower H&Y stage, a shorter disease duration, better balance, less‐severe freezing of gait, and a higher quality of life increased the probability of completing the 1‐year study. However, only the association between balance and freezing remained significant in the multivariate regression model. In the multivariable regression model, a higher score on UPDRS, Part III (more severe motor symptoms), had a higher probability of completing the 1‐year study (HR = 0.95, *P* = 0.002). Excluding participants whose diagnosis changed during study participation (n = 4) and participants who died (n = 2) in the sensitivity analysis did not change our results (Supplementary Material C). The correlation between the independent variables was low, except between motor symptoms and balance impairments (r = 0.65).

**TABLE 2:** Association between participant characteristics and duration of study participation

Characteristic	Univariate analysis	Multivariate analysis n = 151
Demographics		
Age^a^	1.00 [0.97–1.03]	1.00 [0.96–1.05]
Gender (women to men)^b^	1.31 [0.85–2.02]	1.84 [0.96–3.53]
Education level^c^	1.06 [0.89–1.26]	1.12 [0.90–1.39]
Smartphone possession^d^	0.49 [0.28–0.87]*	1.11 [0.31–4.02]
General PD outcomes		
Hoehn & Yahr stage^e^	1.45 [1.09–1.94]*	1.29 [0.76–2.17]
Years since diagnosis^c^	1.04 [1.01–1.08]*	1.03 [0.96–1.08]
UPDRS		
I^f^	1.00 [0.95–1.06]	0.92 [0.84–1.01]
II^f^	1.03 [1.00–1.06]	1.02 [0.96–1.09]
III^g^	1.00 [0.99–1.02]	0.95 [0.92–0.98]**
IV^g^	1.05 [0.99–1.12]	1.06 [0.96–1.16]
Mobility		
Frequency of falls in past year^h^	1.00 [1.00–1.00]	1.00 [0.98–1.00]
Physical activity, sport (LAPAq)^i^	1.00 [1.00–1.01]	1.00 [0.99–1.01]
Physical activity, household (LAPAq)^i^	0.98 [0.96–1.01]	0.99 [0.97–1.01]
Walking speed (6‐meter walking test)^e^	1.05 [0.97–1.13]	0.84 [0.67–1.05]
Balance (miniBEST)^e^	0.96 [0.93–0.99]*	0.90 [0.82–0.99]*
Freezing (new freezing of gait q)^f^	1.03 [1.00–1.05]*	1.04 [1.00–1.08]*
Nonmotor questionnaires		
Fatigue (FSS)^j^	1.01 [0.83–1.23]	1.01 [0.77–1.33]
Anxiety (HADS‐A)^i^	0.98 [0.89–1.09]	0.95 [0.83–1.09]
Depression (HADS‐D)^i^	0.96 [0.83–1.12]	0.97 [0.81–1.17]
Quality of life (PDQ‐39)^j^	1.02 [1.00–1.04]*	1.00 [0.96–1.04]
Cognition (MoCA)^e^	1.02 [0.95–1.10]	1.00 [0.89–1.12]

Hazard ratio (95% confidence interval) is presented. Cox regression analyses were performed with duration of study participation as dependent variable and demographical/clinical characteristics as independent variables. Hazard ratio >1 indicates an increased risk for dropping out for higher scores on the independent variables. Higher scores on independent variables generally indicate worse functioning, except for miniBEST and MoCA, in which higher scores indicate better performance.

For UPDRS, Part I, only questions 7 to 13 were assessed. Number of participants in the univariate analyses is provided.

^a^ n = 185.

^b^ n = 200.

^c^ n = 183.

^d^ n = 198.

^e^ n = 199.

^f^ n = 184.

^g^ n = 196.

^h^ n = 177.

^i^ n = 174.

^j^ n = 182.

* *P*‐value < 0.05.

** *P*‐value < 0.01.

Abbreviations: FSS, fatique severity scale; HADS, hospital anxiety and depression scale; LAPQ, LASA physical activity questionnaire; miniBEST, Mini‐Balance Evaluation Systems Test; MoCA, Montreal Cognitive Assessment; PD, Parkinson's disease; PDQ‐39, parkinson's disease questionnaire‐ 39; UPDRS, Unified Parkinson's Disease Rating Scale.

## Discussion

The aim of this study was to investigate the compliance and user experience of daily use of a remote monitoring system in PwP. The main reason for people to drop out of the study was the wearable sensor itself, including technical problems and a short battery life. However, people who completed the study year experienced similar problems. In our further analyses, we found several factors, that is, demographical or clinical characteristics, that may explain these results.

The proportion of participants who dropped out of our study (42%) is relatively high compared to other studies using remote monitoring systems, which mainly had a shorter follow‐up duration. Dropout rate of those previous studies varied from 0%^12^ to 78%.^16^ There are multiple differences between those studies and the current study, in terms of the study duration, participant characteristics, the study setup and wearable sensor itself, and the research team or support. Here we discuss our findings regarding these aspects in depth and also compare differences in these factors in previous studies.

The first aspect related to compliance is study duration. A consistent finding across studies is that a longer duration of the study is related to lower compliance. Previous studies reported dropout rates of 17%, 27%, and 24% after, respectively, 6 weeks, 13 weeks, and 6 months of follow‐up.^7^, ^8^ At these points in time, our dropout rates were 9%, 19%, and 28%, respectively. We found only 2 studies using a remote monitoring system for ≥1 year.^16^, ^17^ One study included 77 PwP, and after 3 years the dropout rate was 78%.^16^ The other study is the Personalized Parkinson Project^17^ that included 520 PwP; after 2 years only 5% dropped out. This remarkably low dropout rate can be directly attributed to a multifaceted set of measures that were taken to promote patient participation and to determine an optimal compliance. These measures are described in detail in a separate publication.^17^ Besides these measures, this high compliance is likely explained by other factors in the following paragraphs.

A second aspect that might be related to compliance is clinical and demographical characteristics of PwP. Insight into whether these characteristics have an influence on compliance might provide guidance to clinicians in determining which PwP are suitable for monitoring with a wearable sensor and who are not. Although we have to interpret our findings with caution because of the exploratory nature of the analyses, we did identify a few characteristics that might predict study duration. We found that people who already possess a smartphone have a lower probability to drop out of the study. Familiarity—and, subsequently, acquired technical skill—with digital devices might explain this finding. We used smartphone possession in our analysis as a surrogate of technical proficiency. Despite increased interest and new research studies focusing on technological solutions for healthcare problems, there is a remarkable lack of standardized tests to assess technical proficiency and willingness to invest effort to learn and use these new solutions within a patient population.^18^ One such standardized test is the Digital Tool Test; however, its use is still very limited.^19^

Furthermore, participants with more severe motor symptoms had a lower probability of dropping out. This was counterintuitive, because we would expect fewer challenges among PwP with better fine motor skills, such as dealing with the technical issues, but also the general usage of the watch, such as charging. One explanation for this finding could be that technical issues caused more subjective hindrance in daily life in people with less‐severe motor symptoms, and they are expected to be more active during the day. Furthermore, the second explanation could be that those with more severe motor symptoms might be more intrinsically motivated to use a technical application.

Further, participants in our study with better balance and less‐severe freezing were more likely to complete the 1‐year study. Due to our inclusion criterion of at least 1 fall in the 6 months prior to the study inclusion and exclusion of PwP with cognitive or psychiatric impairments that might hinder completion of the study, our results may not be generalizable to all PwP. However, worse balance was a predictor of noncompliance in our study, so PwP in the general PD population (including also nonfallers) might even be more compliant. Besides these motor symptoms, we did not find a relationship between nonmotor symptoms and compliance. We included PwP with a mean age of 69 and a relatively high H&Y stage (61% of participants had a H&Y stage similar to or higher than Stage 3). Compared to other studies, the characteristics of our sample come closer to the real PD population with a mean age of 72 years.^20^ For example, the mean age of the Personalized Parkinson Project cohort^17^ was 62 years. In line with most previous studies,^8^, ^10^ women are slightly underrepresented in this study.

A third aspect that might be related to compliance is the specific device at hand. In the current study, technical problems, including short battery life and nonresponding smartwatches, were a major problem. This short battery life seemed to be setup or device specific, because the Personalized Parkinson Project^17^ had a higher median wearing time of 22.1 h/day^21^ compared to our study (17.5 h/day). Although ideally the median wear time was intended to be ~22 h/day, the median wear time over the entire study period was actually 17.5 h/day, decreasing to 16.1 h in the 12th month. There are several explanations for this. First, 21% of the participants indicated that they did not wear the watch always. Second, 26% of the participants experienced issues that limited the wear time of the watch, a short battery life being the most frequently cited reason. The problem of nonresponding smartwatches was highlighted by the finding that in more than half of the study population the initial watch was replaced at least once. The technical problems of our sensor were mainly due to the secondary purpose of data collection, which will not take place in the eventual implementation in clinical practice. Future studies can, however, build on our lessons learned to enhance participants in their studies, also outside of the field of PD. Besides the technical aspects of the device, esthetical aspects and wearing comfort are important. The latter is especially important, because this was mentioned 12 times as the reason for dropout. Eight watches were replaced during the study due to an allergic reaction or uncomfortable band. This is in line with a review on successful implementation of technology in the management of PD.^22^ Besides the smartwatch, the study setup comprised an online dashboard. Most participants (75 of 98 completed questionnaires) used this online dashboard. However, only 16 participants used this dashboard to gain more insight into their own movement pattern. This low number of users could either reflect a lack of interest in monitoring these personal data or point to low user friendliness with regard to sharing this information. In keeping with this observation, we recently discussed that not all patients are necessarily keen to receive feedback from their wearable sensors, among others, because this would confront them constantly with their disease.^23^

Fourth, there are several measures that the research team could consider to enhance compliance of participants,^17^ such as contact with or support by the research team. That frequent contact moments are important to keep participants involved in the study is also highlighted by 2 findings. First, a large number of participants (n = 71) reported using the online dashboard to report falls as a consequence of the weekly reminder by email. Weekly reminders might be perceived as annoying by participants who had already completed this task. However, given the digital nature of these reminders, they could be easily ignored. Second, participants often approached the research team (available during office hours) regarding technical problems (Supplementary Material B). In addition, we regularly approached the participants to provide technical support when no data were received (Supplementary Material B). These contact moments were rated positively. For future implementation into clinical practice, a contact person or helpdesk may also be needed to assist when technical problems occur. Also, the home visit seemed very useful in this study, because the research team installed the system, resulting in smaller risk of errors. The participants also rated the home visit positively.

This study has several limitations. One is the amount of missing data in baseline questionnaires, resulting in missing values in both demographic and clinical characteristics (15 of 200 participants). Of these 15 participants, a large proportion (10 of 15 participants) were participants who dropped out, which might have influenced these findings. One explanation for more missing data on participants who dropped out is that we were not allowed to remind these participants to return their questionnaires after dropping out of the study. Also, we missed data regarding study experiences (in 18 of 116 participants). However, it seems unlikely that these participants experienced the study differently. A second limitation is that experiences regarding the smartwatch and study were assessed only in participants who completed the study year. Detailed experiences of people who dropped out of the study would be very insightful. However, people had withdrawn their informed consent, and therefore we were allowed to ask for only their reason for dropout and not to collect a full questionnaire for research purposes. Ideally, in informed consents in future studies there should be some space to deliver a short questionnaire about study experiences when withdrawing informed consent.

There are indications that PwP are interested in participating in this type of studies, which is highlighted by the large proportion of participants (75 of 98 participants) who would participate in a future study using a similar watch. Contributing to scientific knowledge of the disease itself and being able to contribute to the development of these kind of sensors are important motivational factors for PwP to participate in research. For future studies using wearable sensors, this will probably still be the case. However, this will not be a motive for PwP during future clinical implementation into actual daily practice, where other motivational factors might be at play. For example, a review of barriers and facilitators to using technology by PwP describes that supporting clinical decision‐making and improving personalized care are facilitators in daily practice.^22^ Furthermore, we assume that the familiarity and thereby the technical skills in the general elderly population, and also in the Parkinson population, will increase in the coming decades. It is also likely that technology, including batteries, will improve. This together could lead to higher compliance rates and usability in the future. Based on our findings, we recommend improving the compliance of a remote monitoring system using a smartwatch that has a longer battery duration. Furthermore, the sensor should have a high wearing comfort. This could be done by offering a fabric watchband and holes in the strap that are not too small, so that they are suitable for PwP whose fine motor skills are affected. Furthermore, future study designs using a remote monitoring system should include fixed and recurring contact moments with and reminders to participants to keep them involved in the study.

In conclusion, the 1‐year compliance of this remote monitor system was moderate, and the user experience was generally good, except battery life and data transfer. We identified demographic and clinical determinants of compliance. We present lessons learned that might be relevant to future studies.

## Author Roles

(1) Research project: A. Conception, B. Organization, C. Execution; (2) Statistical analysis: A. Design, B. Execution, C. Review2 and critique; (3) Manuscript: A. Writing of the first draft, B. Review and critique.

B.R.M.: 1A, 1C, 2A, 2B, 3A

D.H.B.S.: 1A, 2B, 3A

G‐J.V.: 1B, 1C, 2C, 3B

G.V.: 1B, 1C, 2C, 3B

A.E.: 1B, 1C, 2C, 3B

B.R.B.: 2C, 3B

S.K.L.D.: 2C, 3B

N.M.V.: 1A, 1B, 2C, 3B

## Disclosures

**Ethical Compliance Statement**: Bringing Parkinson Care Back Home was approved by the local ethics committee (Commissie Mensgebonden Onderzoek Arnhem‐Nijmegen), reference number 2019‐5845. Participants signed written informed consent before inclusion in the study. We confirm that we have read the journal's position on issues involved in ethical publication and affirm that this work is consistent with those guidelines.

**Funding Sources and Conflicts of Interest**: Bringing Parkinson Care Back Home was funded by the Netherlands Organisation for Health Research and Development. Bart R. Maas and Sirwan K.L. Darweesh are employees of the Proactive and Integrated Management and Empowerment in Parkinson's Disease (PRIME) project, which was funded by the Gatsby Foundation (GAT3676) as well as by the Ministry of Economic Affairs by means of the PPP Allowance made available by the Top Sector Life Sciences & Health to stimulate public–private partnerships. The Center of Expertise for Parkinson & Movement Disorders was supported by a center of excellence grant from the Parkinson's Foundation. The authors declare that there are no conflicts of interest relevant to this work.

**Financial Disclosures for the Previous 12 Months**: Gert‐Jan de Vries, Giulio Valenti, and Andreas Ejupi were employed at Philips Research at the time of study preparation, data collection, data analysis, and drafting of the manuscript. Bastiaan R. Bloem currently serves as editor in chief for the *Journal of Parkinson's Disease*; serves on the editorial board of *Practical Neurology* and Digital Biomarkers; has received honoraria for serving on the scientific advisory boards of AbbVie, Biogen, and UCB; has received fees for speaking at conferences from AbbVie, Zambon, Roche, GE Healthcare, and Bial; and has received research support from the Netherlands Organization for Scientific Research, the Michael J. Fox Foundation, UCB, AbbVie, the Stichting ParkinsonFonds, the Hersenstichting Nederland, the Parkinson's Foundation, Verily Life Sciences, Horizon 2020, the Topsector Life Sciences and Health, the Gatsby Foundation, and the Parkinson Vereniging. Nienke M. de Vries reports grants from the Netherlands Organisation for Health Research and Development (ZonMw), de Hersenstichting Nederland, and the Michael J. Fox Foundation. Sirwan K.L. Darweesh has received funding from the Parkinson's Foundation (PF‐FBS‐2026), ZonMW (09150162010183), ParkinsonNL (P2022‐07 and P2021‐14), the Michael J. Fox Foundation (MJFF‐022767), and the Edmond J. Safra Foundation. Bart R. Maas and Daniël H.B. Speelberg declare that there are no additional disclosures to report.

## Supporting information


**Supplementary Material A.** Reasons for dropout. Participants indicated their reason for dropping out of the study using an open question. Reasons were grouped by theme. One participant was able to give multiple reasons.
**Supplementary Material B.** Experiences of people who completed the study.
**Supplementary Material C.** Sensitivity analyses of the association between participant characteristics and duration of study participation (univariate). Hazard ratio (95% confidence interval) is presented. Cox regression analyses were performed with duration of study participation as dependent variable and demographical/clinical characteristics as independent variables. Hazard ratio >1 indicates an increased risk for dropping out for higher scores on the independent variables. Higher scores on independent variables generally indicate worse functioning, except for miniBEST (Mini‐Balance Evaluation Systems Test) and MoCA (Montreal Cognitive Assessment), in which higher scores indicate better performance. For UPDRS, Part I (Unified Parkinson's Disease Rating Scale, Part I), only questions 7 to 13 were assessed. Number of participants in the univariate analyses.
**Supplementary Material D.** Sociodemographic characteristics of all study participants versus a subgroup of study participants who completed the end‐of‐year questionnaire. Values are mean (standard deviation), unless stated otherwise. Missing values are excluded in calculation percentages.
