# Supplementary material for: Patient Experience and Feasibility of a Remote Monitoring System in Parkinson's Disease
Source: Mov Disord Clin Pract. 2024 Jul 26;11(10):1223–31. doi: 10.1002/mdc3.14169 (PMC11489606; doi:10.1002/mdc3.14169)
Supplement: Supplementary file 1 — Supplementary Material A. Reasons for dropout. Participants indicated their reason for dropping out of the study using an open question. Reasons were grouped by theme. One participant was able to give multiple reasons. Supplementary Material B. Experiences of people who completed the study. Supplementary Material C. Sensitivity analyses of the association between participant characteristics and duration of study participation (univariate). Hazard ratio (95% confidence interval) is presented. Cox regression analyses were performed with duration of study participation as dependent variable and demographical/clinical characteristics as independent variables. Hazard ratio >1 indicates an increased risk for dropping out for higher scores on the independent variables. Higher scores on independent variables generally indicate worse functioning, except for miniBEST (Mini‐Balance Evaluation Systems Test) and MoCA (Montreal Cognitive Assessment), in which higher scores indicate better performance. For UPDRS, Part I (Unified Parkinson's Disease Rating Scale, Part I), only questions 7 to 13 were assessed. Number of participants in the univariate analyses. Supplementary Material D. Sociodemographic characteristics of all study participants versus a subgroup of study participants who completed the end‐of‐year questionnaire. Values are mean (standard deviation), unless stated otherwise. Missing values are excluded in calculation percentages. [file MDC3-11-1223-s001.docx]

**Supplementary material**

**Patient experience and feasibility of a remote monitoring system in Parkinson’s disease**

Bart R. Maas^1^, MSc, Daniël H.B. Speelberg^1^, MD, Gert-Jan de Vries^2^, PhD, Giulio Valenti^2^, PhD, Andreas Ejupi^2^, PhD, Bastiaan R. Bloem^1^, MD, PhD, FRCPE, Sirwan K.L. Darweesh^1^, MD, PhD, Nienke M. de Vries^1^, PhD

^1^ Department of Neurology, Radboud University Medical Center, Donders Institute for Brain, Cognition and Behavior, Center of Expertise for Parkinson and Movement Disorders, Nijmegen, The Netherlands

^2^ Philips Research - Healthcare, Eindhoven, the Netherlands.

**Corresponding author:** Nienke M. de Vries, PhD

Department of Neurology, Donders Institute for Brain, Cognition and Behavior, Radboud University Medical Center, P.O. Box 9101, 6500 HB, Nijmegen, The Netherlands. E-mail: Nienke.DeVries@radboudumc.nl

**Supplementary material A.** Reasons for drop-out

| **Reason** | **Frequency reason** |
| --- | --- |
| **Smartwatch related**  Technical problems  Battery life  Wearing comfort  Too complicated in use  Watch lacks necessary functions^1^ | 23  17  12  5  4 |
| **Research burden related**  Research too burdensome  Decrease in motivation  Poor communication research team | 15  8  1 |
| **Health related**  Decline in health (and mobility)  Additional health problems besides PD  Deceased | 11  4  2 |
| **Miscellaneous**  Change of diagnosis to parkinsonism  Personal external circumstances  Unknown | 2  6  8 |

^1^Features lacking included alarm clocks for medication intake and fall detection.

Participants indicated their reason for dropping out of the study using an open question. Reasons were grouped by theme. One participant was able to give multiple reasons.

**Supplementary material B.** Experiences of people that completed the study

| **Multiple-choice items** | **Frequency of answers** |
| --- | --- |
| **How did you experience the home visit at the start of the study?**  Very pleasant  Nice  Neutral  Not pleasant  Not pleasant at all | 32  56  10  0  0 |
| **How did you experience the contact with the research team during the research?**  Very pleasant  Nice  Neutral  Not pleasant  Not pleasant at all | 23  59  16  0  0 |
| **Have you ever had to contact the research team by yourself due to technical problems in the past year?**  Yes, once  Yes, several times  No | 30  54  14 |
| **What was the nature of the problem? (multiple answers possible)**  The display of the watch only gave a black image  The watch's display stopped responding to swipe gestures  The data from the watch was not forwarded  The charger of the watch was defective  It was no longer enough to charge the watch twice a day  The time and/or date of the watch had been changed  The Wifi hub only gave a black image  The charger of the WiFi hub was defective  It was not possible to log in to the online dashboard (WebApp)  It was not possible to (correctly) complete the digital fall questionnaire  My daily activities were not or only partially visible on the online dashboard  I had a question about my data regarding daily activity on the online dashboard  Other | 16  13  46  11  24  40  3  2  10  10  7  10  11 |
| **Was the research team able to solve this problem?**  Yes, every time  Yes, but not every time  Yes, but it took a lot of time and/or effort  No, the watch needed to be replaced  No, the Wifi Hub needed to be replaced | 58  6  1  18  1 |
| **In the past year, has the research team contacted you about not receiving data from your watch?**  Yes, once  Yes, several times  No | 34  36  28 |
| **Was the research team able to solve this problem?**  Yes, every time  Yes, but not every time  Yes, but it took a lot of time and/or effort  No, the watch needed to be replaced  No, the Wifi Hub needed to be replaced | 55  0  1  14  0 |
| **Has your watch ever been replaced by a new one?**  Yes, once  Yes, several times  No | 47  5  46 |
| **Has your Wifi Hub ever been replaced by a new Wifi Hub?**  Yes, once  Yes, several times  No | 5  2  91 |
| **Were you used to wearing a watch prior to participating in this study?**  Yes  No | 80  18 |
| **Do you think the watch is the right way to process a sensor for data collection?**  Yes, the sensor is well processed in this way  No, I prefer to wear a sensor necklace  No, I prefer to wear a different type of sensor | 87  5  6 |
| **What do you think about the size of the watch?**  Too big  Fine  Too small | 31  67  0 |
| **What do you think about the format of the display?**  The size of the text and icons are easy to read/see  The size of the text and icons are acceptable  The size of the text and icons are too small | 53  31  14 |
| **What do you think of the look of the watch?**  I like it very much  I think it is beautiful  I am fine with it  I do not think it's beautiful  I don't like it at all | 9  13  50  17  9 |
| **How do you experience wearing the watch strap?**  It wears comfortably  It's not comfortable to wear | 67  31 |
| **Can you indicate why you experience wearing this strap as unpleasant? (multiple answers possible)**  It is sticky/sweaty  It itches  It gives me a rash  The strap is too stiff  The strap is difficult to put on  Other | 11  5  2  13  15  7 |
| **Have you worn the watch both day and night?**  Yes  No, only during the day  Other | 77  16  5 |
| **At what times did you charge the watch?**  When it indicated it was empty  At fixed times  Other | 43  45  10 |
| **Have you experienced any issues limiting your wearing of the watch?**  Yes  No | 25  73 |
| **What were those problems? (multiple answers possible)**  The short battery life  The disabled additional functions  The weight of the watch  The (fastening of the) watch strap  Lighting up the screen during the night  Other | 18  3  0  7  2  7 |
| **What were your reasons for wearing the watch? (multiple answers possible)**  Use it like a normal watch  The insights into daily activity on the online dashboard  The contribution to scientific research  The contribution to the development of this type of watches  Other | 30  7  91  24  1 |
| **Have you used the online dashboard (WebApp) to report falls?**  Yes  Sometimes  Never  No, I used paper fall diary | 65  10  16  7 |
| **Have you experienced problems that limited the use of this website?**  Yes  No | 11  80 |
| **What were your reasons for using the online dashboard? (multiple answers possible)**  The weekly reminder by email  The insights into my own movement pattern  The insights into previously reported falls  Other | 71  16  11  11 |
| **Have you used the data from the online dashboard to adjust your lifestyle?**  Yes, the data of my daily activities  Yes, the data about my falls  No | 3  4  84 |
| **During the study, did you consider stopping your participation in this study?**  Yes  No | 19  79 |
| **Would you participate in a future study using a similar watch (possibly using a similar online dashboard)?**  Yes  No | 75  23 |

**Supplementary material C.** Sensitivity analyses on the association between participant characteristics and duration of study-participation (univariate)

|  | **All participants** | **Without participants in whom diagnosis changed or who died** |
| --- | --- | --- |
| **Demographics**  Age^a^  Gender (women to men)^b^  Education level^c^  Smartphone possession^d^ | 1.00 [0.97-1.03]  1.31 [0.85-2.02]  1.06 [0.89-1.26]  0.49 [0.28-0.87]* | 0.99 [0.96-1.02]  1.37 [0.87-2.14]  1.09 [0.91-1.31]  0.51 [0.28-0.93]* |
| **General PD outcomes**  Hoehn & Yahr stage^e^  Years since diagnosis^c^  UPDRS  I^f^  II^f^  III^g^  IV^g^ | 1.45 [1.09-1.94]*  1.04 [1.01-1.08]*  1.00 [0.95-1.06]  1.03 [1.00-1.06]  1.00 [0.99-1.02]  1.05 [0.99-1.12] | 1.38 [1.03-1.86]*  1.04 [1.00-1.08]*  1.01 [0.96-1.07]  1.03 [1.00-1.06]  1.00 [0.98-1.02]  1.05 [0.99-1.12] |
| **Mobility**  Frequency of falls in past year^h^  Physical activity, sport (LAPAq)^i^  Physical activity, household (LAPAq)^i^  Physical capacity (Six meter walking test)^e^  Balance (miniBEST)^e^  Freezing (new freezing of gait q)^f^ | 1.00 [1.00-1.00]  1.00 [1.00-1.01]  0.98 [0.96-1.01]  1.05 [0.97-1.13]  0.96 [0.93-0.99]*  1.03 [1.00-1.05]* | 1.00 [1.00-1.00]  1.00 [1.00-1.01]  0.98 [0.96-1.00]  1.04 [0.96-1.12]  0.96 [0.93-1.00]*  1.03 [1.00-1.05]* |
| **Non-motor questionnaires**  Fatigue (FSS)^j^  Anxiety (HADS-A)^i^  Depression (HADS-D)^i^  Quality of Life (PDQ-39)^j^  Cognition (MoCA)^e^ | 1.01 [0.83-1.23]  0.98 [0.89-1.09]  0.96 [0.83-1.12]  1.02 [1.00-1.04]*  1.02 [0.95-1.10] | 1.01 [0.83-1.23]  0.97 [0.88-1.08]  0.95 [0.81-1.10]  1.02 [1.00-1.04]*  1.04 [0.96-1.13] |

Hazard ratio [95% confidence interval] is presented. Cox regression analyses were performed with duration of study-participation as dependent variable and demographical/clinical characteristics as independent variables. Hazard ratio >1 indicates an increased risk for dropping out for higher scores on the independent variables. Higher scores on independent variables generally indicate worse functioning, except for miniBEST and MoCA, in which higher scores indicate better performance.

For the UPDRS-I, only questions 7-13 were assessed. Number of participants in the univariate analyses:

*^a^n=185; ^b^n=200; ^c^n=183; ^d^n=198; ^e^n=199; ^f^n=184; ^g^n=196; ^h^n=177; ^i^n=174; ^j^n=182*

**p-value < 0.05; **p-value <0.01*

**Supplementary material D.** Sociodemographic characteristics of all study participants vs. a subgroup of study participants who completed the end-of-year questionnaire

|  | **Total study cohort**  n=200 | **Participants who completed end-of-year questionnaire**  n=98 |
| --- | --- | --- |
| **Age***, years* | 68.7 (7.4)*^a^* | 68.4 (7.3) |
| **Women***, n (%)* | 74 (37) | 35 (36) |
| **Education***, n (%)^b^*  None  Secondary school  Lower vocational education  Secondary vocational education  Higher professional education  University | 2 (1)  28 (15)  17 (9)  38 (21)  67 (37)  31 (17) | 1 (1)  16 (17)  11 (11)  19 (20)  34 (35)  15 (16) |
| **Disease characteristics**  Years since diagnosis*^b^*  Hoehn & Yahr scale*, n (%)^c^*  I  II  III  IV  V | 9.1 (5.8)  12 (6)  65 (33)  94 (47)  27 (14)  1 (<1) | 8.1 (5.5)  9 (9)  34 (35)  48 (49)  7 (7)  0 (0) |

Values are mean (standard deviation), unless stated otherwise. Missing values are excluded in calculation percentages. Missing data :

^a^Missing data of 15 participants. ^b^Missing data of 17 participants in the total cohort; missing data of 2 participants who completed end-of-year questionnaire. ^c^Missing data of 1 participant in the total cohort.
